# Supplementary material for: Serum Metabolites Responding in a Dose-Dependent Manner to the Intake of a High-Fat Meal in Normal Weight Healthy Men Are Associated with Obesity
Source: Metabolites. 2021 Jun 16;11(6):392. doi: 10.3390/metabo11060392 (PMC8233812; doi:10.3390/metabo11060392)
Supplement: Supplementary file 1 [file metabolites-11-00392-s001.zip › metabolites-1257117-supplementary.pdf]

Serum metabolites responding in a dose-dependent manner to the intake of a high-fat meal in normal weight healthy men are associated with obesity

SUPPLEMENTAL TABLE S1 Characterization of the fasting (t0) and postprandial (iAUC 0-6h) values of NW (n = 15) and OB (n = 17) subjects for the 135 dose-dependent features identified in the NW group after ingestion of 500, 1000, and 1500 kcal of the HF-meal[1]

No	Identification	LI	Rt	Mass m/z	Median t0					Median iAUC NW							Median iAUC OB						
			min	Da	NW	sem	OB	sem	p	A	sem	B	sem	C	sem	FDR	A	sem	B	sem	C	sem	FDR
66	No identification	4	3.36	102.046334	0.99	0.11	1.14	0.09	0.02	0.23	0.37	1.52	0.25	0.96	0.40	0.001	0.32	0.28	0.50	0.30	1.11	0.60	0.176
693	Proline	1	1.06	116.070699	0.76	0.06	0.90	0.07	0.07	0.47	0.16	1.61	0.17	1.68	0.21	0.000	0.12	0.15	0.68	0.15	1.05	0.20	0.013
781	Valine	1	1.23	118.086402	0.79	0.03	0.86	0.04	0.01	0.38	0.10	1.33	0.11	1.10	0.18	0.001	0.08	0.10	0.60	0.10	0.84	0.11	0.000
858	Homoserine or threonine	3	0.99	120.065394	1.11	0.09	1.27	0.06	0.01	0.47	0.24	1.88	0.13	1.74	0.19	0.000	0.24	0.23	0.02	0.20	1.34	0.29	0.021
867	No identification	4	3.36	120.080841	0.95	0.11	1.27	0.10	0.01	0.18	0.47	1.89	0.31	1.26	0.48	0.000	0.59	0.34	0.54	0.28	1.64	0.65	0.126
1006	2-Hydroxybenzaldehyde	2	1.26	123.043976	0.99	0.03	1.21	0.03	0.00	-0.06	0.12	0.72	0.17	1.01	0.15	0.000	-0.17	0.12	0.12	0.14	0.69	0.15	0.000
1419	(2E,4E)-Hexa-2,4-dienoic acid	2	0.85	130.086113	0.95	0.09	1.32	0.07	0.01	1.05	0.31	2.57	0.30	2.57	0.37	0.000	0.92	0.23	1.35	0.26	2.35	0.43	0.120
1474	No identification	4	3.36	131.049007	1.19	0.12	1.51	0.09	0.06	0.60	0.30	1.53	0.26	1.28	0.44	0.000	0.40	0.28	0.29	0.23	1.84	0.54	0.133
1544	Creatine	1	1.04	132.077166	0.43	0.04	0.74	0.10	0.00	1.86	0.15	2.99	0.29	3.97	0.40	0.000	1.65	0.28	3.28	0.31	4.45	0.41	0.000
1551	Triethanolamine	2	1.25	132.102095	0.67	0.04	0.75	0.04	0.21	1.16	0.16	1.68	0.20	1.94	0.30	0.000	0.45	0.13	1.17	0.17	1.45	0.17	0.000
1558	No identification	4	1.26	132.168815	0.60	0.07	0.69	0.08	0.25	0.07	0.38	1.84	0.56	2.66	0.51	0.001	0.28	0.43	1.03	0.44	1.77	0.58	0.100
1588	No identification	4	1.26	133.031737	0.91	0.03	1.00	0.03	0.25	0.07	0.14	1.48	0.18	1.89	0.18	0.000	0.00	0.10	0.35	0.11	0.84	0.19	0.000
1659	Alanine	1	0.97	134.018646	0.95	0.06	1.23	0.05	0.02	-0.28	0.25	0.36	0.15	0.69	0.22	0.003	-0.20	0.22	-0.14	0.23	-0.01	0.21	0.569
1797	Benzofuran	2	1.26	136.075706	0.97	0.03	1.17	0.04	0.00	0.04	0.15	0.75	0.16	0.98	0.15	0.000	-0.19	0.13	0.32	0.15	0.64	0.15	0.000
2518	Phenylpropiolic acid	2	1.26	147.044001	1.01	0.04	1.19	0.04	0.00	-0.12	0.14	1.08	0.17	0.84	0.17	0.000	-0.20	0.17	0.28	0.15	0.43	0.14	0.000
2548	Lysine	1	0.86	147.112601	0.97	0.09	1.30	0.07	0.00	0.84	0.35	2.63	0.31	2.62	0.39	0.000	0.96	0.23	1.14	0.25	2.48	0.40	0.070
2694	No identification	4	0.95	149.992845	1.70	0.20	1.76	0.15	0.33	-1.48	0.98	0.69	0.50	1.73	0.53	0.000	-0.25	0.41	-0.50	0.64	-0.32	0.43	0.235
2696	No identification	4	0.95	150.013489	1.17	0.09	1.21	0.05	0.24	0.57	0.37	1.47	0.19	1.71	0.23	0.001	0.55	0.18	0.29	0.22	0.90	0.27	0.068
2704	No identification	4	1.26	150.058349	0.87	0.03	0.94	0.03	0.50	0.26	0.14	1.68	0.19	2.28	0.18	0.000	0.05	0.11	0.51	0.12	0.98	0.20	0.000
2960	2,3-Butanediol	2	2.42	154.083594	0.74	0.09	0.96	0.08	0.00	-0.10	0.53	1.97	0.28	2.52	0.22	0.000	0.84	0.30	1.10	0.33	1.41	0.37	0.516
2961	No identification	4	1.25	154.083752	0.69	0.04	0.70	0.05	0.47	0.75	0.15	1.57	0.15	1.63	0.22	0.000	0.51	0.11	0.66	0.12	0.98	0.17	0.049
3575	No identification	4	0.97	164.028974	0.94	0.06	0.99	0.04	0.14	0.18	0.19	1.67	0.21	1.53	0.17	0.000	0.31	0.18	0.45	0.17	0.70	0.15	0.183
3632	No identification	4	1.26	165.054484	1.06	0.04	1.24	0.04	0.02	-0.09	0.17	0.70	0.15	1.14	0.17	0.000	-0.16	0.13	0.24	0.17	0.54	0.15	0.000
3681	Gentiatibetine	2	3.36	166.086114	0.96	0.12	1.26	0.10	0.02	0.45	0.49	1.76	0.30	1.32	0.49	0.000	0.54	0.34	0.45	0.27	1.81	0.65	0.112
3708	No identification	4	3.36	166.161719	0.99	0.09	1.09	0.09	0.08	0.24	0.37	1.25	0.28	1.47	0.52	0.000	0.85	0.59	0.31	0.38	0.09	0.59	0.722
3852	No identification	4	3.36	168.091325	1.05	0.11	1.30	0.11	0.01	-0.35	0.44	1.47	0.22	0.85	0.41	0.001	0.34	0.33	-0.03	0.26	1.10	0.54	0.153
3902	No identification	4	0.90	169.094326	0.97	0.08	1.25	0.09	0.01	0.69	0.30	2.27	0.30	2.85	0.31	0.000	0.80	0.34	1.05	0.21	2.56	0.42	0.059
4189	Glutamic gamma-semialdehyde	2	1.16	173.091622	0.44	0.05	0.50	0.05	0.14	2.08	0.28	4.12	0.65	4.41	0.98	0.000	1.39	0.49	3.14	0.41	3.94	0.60	0.000
4310	N-Tigloylglycine	2	1.13	175.107448	0.50	0.31	0.50	0.07	0.65	0.80	0.08	1.44	0.33	2.45	0.29	0.000	0.87	0.09	1.47	0.19	1.83	0.21	0.000
4315	Arginine	1	0.95	175.118639	0.95	0.08	1.31	0.07	0.00	0.62	0.28	2.18	0.30	2.39	0.24	0.000	1.06	0.19	0.63	0.23	2.16	0.36	0.010
4389	Asparagine	2	0.95	177.024234	1.16	0.07	1.16	0.04	0.81	0.48	0.32	1.23	0.19	1.59	0.24	0.000	0.39	0.15	-0.20	0.21	0.58	0.27	0.299
4408	Ornithine	2	0.90	177.060617	1.08	0.07	1.17	0.06	0.07	1.04	0.29	2.49	0.29	2.22	0.31	0.000	1.08	0.16	1.12	0.15	2.02	0.38	0.089
4671	No identification	4	3.46	181.095883	0.60	0.04	0.56	0.03	0.72	-0.27	0.20	0.52	0.12	0.63	0.15	0.000	0.46	0.16	0.12	0.13	0.61	0.16	0.398
4740	Tyrosine	2	1.26	182.081165	0.98	0.04	1.19	0.04	0.00	-0.07	0.15	1.11	0.17	1.59	0.19	0.000	-0.17	0.16	0.40	0.15	0.68	0.15	0.000
4747	No identification	4	1.25	182.105562	0.54	0.03	0.61	0.04	0.35	1.22	0.14	1.81	0.20	2.07	0.23	0.000	0.46	0.14	1.30	0.14	1.67	0.17	0.000
4896	A alpha C	2	2.29	184.085796	0.80	0.08	1.06	0.09	0.00	-0.88	0.41	1.23	0.22	2.13	0.34	0.000	0.28	0.36	0.48	0.40	0.67	0.50	0.244
5270	Homoarginine	1	0.97	189.134128	1.03	0.10	1.32	0.11	0.04	-0.07	0.20	0.85	0.37	0.84	0.14	0.000	0.23	0.22	-0.31	0.34	0.04	0.31	0.265

No	Identification	LI	Rt	Mass m/z	Median t0					Median iAUC NW							Median iAUC OB						
			min	Da	NW	sem	OB	sem	p	A	sem	B	sem	C	sem	FDR	A	sem	B	sem	C	sem	FDR
6278	Cys-Asp-Arg	2	13.2	205.595142	0.41	0.07	0.39	0.07	0.88	2.61	0.43	4.70	0.39	4.44	0.60	0.003	1.14	0.34	3.00	0.31	3.01	0.47	0.000
6369	2-Methylcitric acid	2	0.88	207.050182	1.12	0.12	1.56	0.13	0.00	0.24	0.70	4.13	0.66	3.47	0.51	0.001	0.85	0.53	1.68	0.70	1.41	0.78	0.746
6672	Dihydroxyfumaric acid	2	1.04	212.016811	0.53	0.06	0.80	0.10	0.02	1.87	0.20	3.31	0.39	4.41	0.41	0.000	1.38	0.30	3.54	0.31	4.34	0.40	0.000
6833	Met-Gln-Lys	2	13.2	214.60049	0.35	0.07	0.25	0.08	0.24	3.14	0.42	6.24	0.72	4.53	0.82	0.000	1.50	0.30	3.69	0.50	3.48	0.48	0.000
6992	1-4 Benzo-quinone	2	0.86	217.049101	0.91	0.06	1.00	0.05	0.29	0.97	0.40	2.47	0.29	2.93	0.22	0.000	0.84	0.27	1.70	0.31	1.63	0.32	0.129
7084	2-(2-Butoxyethoxy) acetic acid	2	2.80	218.138199	0.83	0.07	0.93	0.09	0.14	0.00	0.39	1.43	0.27	1.24	0.37	0.000	0.52	0.38	0.38	0.30	1.33	0.52	0.249
7137	1-(2-Thienyl)-1-heptanone	2	0.95	219.082367	0.95	0.07	1.29	0.07	0.00	0.58	0.28	2.15	0.30	1.96	0.17	0.000	0.95	0.19	0.54	0.22	1.65	0.33	0.019
7141	Glu-Ala	2	1.25	219.097095	0.93	0.07	1.17	0.05	0.05	-0.24	0.19	0.65	0.21	0.97	0.27	0.000	-0.28	0.21	-0.12	0.13	0.54	0.25	0.114
7796	N,N'-Diallyl-2,3-dihydroxysuccinamide	2	1.19	229.117813	0.73	0.07	0.65	0.11	0.26	0.87	0.23	1.40	0.30	1.35	0.26	0.000	0.56	0.22	1.38	0.62	1.49	0.34	0.000
7876	Coriandrin	2	0.86	231.064696	0.85	0.07	1.14	0.07	0.01	0.67	0.30	2.26	0.26	2.05	0.29	0.000	0.44	0.17	1.27	0.25	1.67	0.37	0.053
7904	No identification	4	1.02	231.14461	0.86	0.06	0.73	0.06	0.63	0.46	0.22	2.37	0.30	2.16	0.23	0.000	0.86	0.24	1.15	0.19	1.12	0.24	0.593
7980	No identification	4	1.06	232.950335	1.14	0.05	1.03	0.06	0.16	-0.13	0.23	-0.46	0.30	-1.09	0.23	0.001	-0.21	0.29	0.56	0.31	-0.12	0.32	0.171
8278	Salithion	2	1.06	238.991147	0.70	0.09	0.61	0.11	0.58	1.29	5.51	3.35	0.39	2.92	0.30	0.000	0.74	0.53	2.45	0.28	2.82	0.96	0.020
8282	2,3-Naphtalendicarboxylic acid	2	0.88	239.030961	0.80	0.07	0.92	0.07	0.86	1.14	0.36	1.97	0.29	3.14	0.35	0.002	0.81	0.31	1.68	0.31	1.31	0.35	0.288
8488	Tetramethyluric acid	2	1.04	242.12423	0.91	0.14	0.74	0.09	0.63	0.54	0.39	1.55	0.22	1.92	0.57	0.004	0.19	0.34	0.10	0.25	0.80	0.26	0.761
8719	Isobutyl octanoate	2	3.10	245.149088	0.90	0.10	0.94	0.08	0.50	0.38	0.47	2.00	0.32	1.63	0.44	0.000	0.62	0.34	1.50	0.49	1.97	0.61	0.046
8826	No identification	4	3.22	247.128294	0.95	0.08	1.25	0.09	0.00	-0.37	0.34	1.85	0.19	1.32	0.29	0.000	0.30	0.28	0.31	0.23	1.15	0.39	0.086
8923	Creatinine	2	1.18	249.107463	0.84	0.05	0.91	0.05	0.41	0.20	0.12	0.99	0.20	0.97	0.22	0.001	0.13	0.12	0.73	0.18	0.85	0.14	0.033
9144	Palythazine	2	3.08	253.117819	1.07	0.12	1.68	0.23	0.00	0.09	0.61	1.72	0.36	1.28	0.29	0.000	1.00	0.34	0.03	0.48	1.32	0.70	0.205
9539	2-((3-Methyl-5-oxo-4,5-dihydro-1H-pyrazol-4-yl))2-thienyl)methyl)malononitrile	2	0.90	259.063581	1.09	0.09	1.54	0.09	0.00	1.14	0.47	2.54	0.49	4.07	0.36	0.000	1.00	0.35	0.80	0.30	3.08	0.55	0.035
9624	Hexanoylcarnitine	2	5.95	260.185106	1.02	0.09	1.10	0.08	0.17	-0.89	0.37	-0.65	0.34	-1.78	0.44	0.004	-0.64	0.33	-0.98	0.28	-1.36	0.38	0.551
9651	No identification	4	9.31	260.620872	0.00	0.05	0.00	0.15	0.79	1.81	0.97	4.63	1.03	7.08	1.46	0.000	2.74	1.35	3.83	2.39	3.96	3.03	0.012
9703	3-Hydroxydodecanoic acid	2	4.26	261.143884	0.84	0.05	0.98	0.07	0.00	0.84	0.16	2.33	0.22	2.88	0.38	0.000	0.77	0.30	1.35	0.23	2.29	0.33	0.000
9826	Benzyl-4-methyl-1,3-dihydro-1,4-benzodiazepine	2	1.04	263.119707	1.07	0.11	0.95	0.07	0.95	-0.04	0.47	2.02	0.36	1.70	0.50	0.005	0.79	0.47	1.34	0.33	1.81	0.47	0.217
9843	No identification	4	1.25	263.196119	0.48	0.05	0.65	0.08	0.11	1.87	0.20	2.70	0.45	4.32	0.50	0.000	1.19	0.30	2.53	0.34	3.22	0.37	0.000
10249	No identification	4	1.04	269.975144	0.64	0.06	0.82	0.07	0.01	1.09	0.21	2.14	0.31	2.84	0.31	0.000	1.14	0.29	1.55	0.21	2.18	0.34	0.000
10564	3-Mercaptohexyl hexanoate	2	8.82	274.182642	0.91	0.06	1.13	0.09	0.02	-0.21	0.23	-0.25	0.17	-1.17	0.23	0.003	-0.26	0.26	-0.60	0.26	-1.04	0.24	0.005
10669	Glu-Gln	2	1.06	276.11839	1.15	0.09	1.23	0.09	0.35	-0.24	0.32	0.68	0.35	1.40	0.28	0.000	0.24	0.25	-0.15	0.26	-0.22	0.42	0.616
10855	No identification	4	1.26	279.100326	0.78	0.03	0.79	0.03	0.21	0.54	0.14	1.14	0.20	1.34	0.22	0.000	0.10	0.11	0.71	0.12	0.97	0.13	0.000
11346	No identification	4	1.25	286.114055	1.14	0.11	1.36	0.09	0.06	0.46	0.32	0.06	0.33	-1.02	0.30	0.004	-0.29	0.43	0.21	0.31	0.13	0.27	0.227
11350	N-Nonanoyl-homoserine lactone	2	1.23	286.138722	0.50	0.18	0.46	0.10	0.58	0.53	0.62	3.19	0.60	3.53	0.93	0.000	1.31	0.41	1.99	0.51	2.28	0.94	0.111
11353	Erysopine	2	11.3	286.143568	0.46	0.24	0.71	0.17	0.83	0.70	0.31	1.63	0.98	1.09	0.42	0.002	0.05	0.22	1.18	0.28	0.83	0.34	0.000
11474	Cetraxate HCl	2	11.2	288.159977	0.48	0.05	0.51	0.18	0.33	2.08	0.35	2.99	0.34	3.15	0.34	0.003	1.53	0.39	2.47	0.60	2.76	0.37	0.030
11490	Valprolycarnitine	2	7.71	288.216447	1.00	0.15	0.98	0.12	0.43	-1.24	0.57	-1.60	0.59	-3.00	0.81	0.002	-0.84	0.55	-1.51	0.50	-1.35	0.68	0.357
12321	2,6-Dimethylheptanoyl carnitine	2	8.02	302.23198	1.01	0.19	0.55	0.09	0.14	-0.93	0.53	-0.37	0.60	-2.69	0.58	0.003	-0.51	0.18	-0.53	0.20	-0.69	0.22	0.838
12357	Pyrogallol-2-O-gluconide	2	3.37	303.070362	0.14	0.06	0.08	0.04	0.21	1.41	0.40	3.49	1.02	4.20	0.86	0.000	1.87	0.37	3.73	0.63	6.25	1.08	0.000
12456	Indole-3-carboxilic acid-O-sulphate	2	4.19	305.020622	1.13	0.08	1.40	0.08	0.00	-0.01	0.46	1.96	0.37	1.61	0.35	0.000	0.56	0.32	-0.31	0.34	0.85	0.53	0.055
12571	No identification	4	1.26	307.101639	1.06	0.08	1.08	0.08	0.86	0.10	0.36	-0.90	0.36	-1.34	0.30	0.005	-0.90	0.29	-1.17	0.43	-1.16	0.33	0.436

No	Identification	LI	Rt	Mass m/z	Median t0					Median iAUC NW							Median iAUC OB						
			min	Da	NW	sem	OB	sem	p	A	sem	B	sem	C	sem	FDR	A	sem	B	sem	C	sem	FDR
12793	4-(4-Methanesulfonylphenyl)-3-(4-methylphenyl)-5H-furan-2-one	2	1.06	311.073499	0.71	0.07	0.90	0.10	0.01	0.58	0.27	2.83	0.40	3.66	0.33	0.000	0.20	0.26	0.95	0.25	2.06	0.28	0.000
12804	1alpha,15-Dihydroxymaraseme	2	3.51	311.123044	0.94	0.12	1.31	0.12	0.01	-0.13	0.53	1.74	0.24	1.66	0.36	0.000	0.14	0.37	0.09	0.36	0.98	0.57	0.202
12846	20-Dihydrodydrogeterone	2	10.7	311.272752	0.21	0.05	0.26	0.08	0.76	3.22	0.60	3.99	0.92	5.49	0.64	0.000	2.44	0.58	4.38	1.13	5.83	1.43	0.000
12879	2-trans,4-cis-Decadienoylcarnitine	2	8.07	312.216353	0.99	0.12	1.01	0.10	0.72	-1.48	0.41	-0.88	0.49	-2.40	0.51	0.004	-1.20	0.43	-1.29	0.35	-1.76	0.39	0.588
12916	4-Hydroxy-5-(3'-hydroxyphenyl)-valeric acid-3'-O-sulphate	2	0.9	313.035204	1.28	0.09	1.44	0.09	0.03	1.39	0.47	3.53	0.50	4.12	0.41	0.000	1.98	0.34	2.69	0.34	3.13	0.62	0.090
12953	No identification	4	1.25	313.17525	0.59	0.04	0.79	0.07	0.03	1.14	0.23	2.23	0.27	2.78	0.38	0.000	0.42	0.19	2.02	0.24	2.55	0.30	0.000
13313	Glutamyl-methionine	2	1.06	319.119607	0.31	0.04	0.41	0.05	0.04	-0.09	0.13	0.69	0.13	0.96	0.13	0.000	-0.03	0.17	0.18	0.20	0.11	0.23	0.824
13414	Salicylate-sodium	2	0.88	321.033866	0.79	0.07	1.13	0.07	0.02	0.82	0.33	2.23	0.35	2.39	0.38	0.002	0.76	0.26	1.27	0.29	1.50	0.27	0.145
13477	No identification	4	0.99	322.110118	1.40	0.12	1.52	0.08	0.07	0.52	0.25	1.88	0.26	2.14	0.26	0.000	0.55	0.19	0.07	0.28	1.31	0.28	0.002
13598	Flumequine	2	12.6	325.096516	1.47	0.15	1.32	0.16	0.65	-0.82	0.81	-0.89	0.58	-3.41	0.73	0.004	-0.03	0.53	-0.09	0.76	-1.63	0.51	0.021
13672	Clemizole	2	14.9	326.141885	2.03	0.29	2.70	0.29	0.04	-4.61	1.46	-7.11	1.28	-7.17	1.38	0.000	-5.83	1.13	-8.61	1.51	-5.23	1.47	0.145
13726	Lamivudine-monophosphate	2	0.90	327.05083	1.30	0.10	1.75	0.09	0.00	1.19	0.50	3.47	0.47	4.16	0.40	0.000	1.50	0.29	1.16	0.31	2.86	0.67	0.075
13928	No identification	4	1.06	329.929954	0.23	0.08	0.40	0.11	0.08	1.15	0.44	2.57	0.42	2.46	0.62	0.005	0.82	0.39	2.76	0.41	2.80	0.46	0.000
13980	No identification	4	1.26	331.131401	0.67	0.03	0.78	0.04	0.35	0.72	0.20	1.86	0.25	2.35	0.32	0.000	0.01	0.17	1.20	0.15	1.94	0.21	0.000
14126	Allantoin	1	1.06	159.051815	0.63	0.05	0.79	0.10	0.01	1.56	3.15	3.35	0.24	3.99	0.30	0.000	1.55	0.31	2.73	0.38	3.94	0.71	0.000
14182	No identification	4	1.06	335.050406	0.82	0.06	0.80	0.06	0.50	0.99	0.26	2.41	0.34	1.88	0.23	0.000	0.40	0.29	1.08	0.31	1.65	0.31	0.013
14452	No identification	4	10.7	339.266784	0.47	0.08	0.58	0.36	0.98	1.80	0.26	2.49	0.40	3.52	0.36	0.000	1.33	0.71	2.35	0.73	3.32	1.07	0.041
14622	Trans-2-Dodecenoylcarnitine	2	9.76	342.263102	1.02	0.18	1.23	0.20	0.02	-1.30	0.74	-0.70	0.72	-2.86	1.06	0.005	-1.44	1.09	-2.20	0.76	-1.97	1.10	0.474
14692	N-[3-(3-Methoxyphenyl)-4-oxochromen-2-yl]furan-2-carboxamide	2	0.99	344.091955	1.10	0.10	1.25	0.06	0.02	0.34	0.28	1.82	0.28	1.76	0.25	0.000	0.82	0.23	0.34	0.24	1.35	0.25	0.043
14785	MG(i-17:0/0:0/0:0) 1-15-Methylhexadecanoyl-sn-glycerol	2	8.79	345.299045	0.71	0.12	0.56	0.12	0.09	0.49	0.43	-0.49	0.33	-0.83	0.31	0.003	0.40	0.24	-0.07	0.44	-0.14	0.36	0.737
15392	Triazophos	2	9.31	358.033688	1.25	0.25	0.64	0.24	0.48	0.41	0.32	0.04	0.31	-0.79	0.38	0.000	-0.08	0.35	-0.24	0.27	0.07	0.33	0.616
16481	No identification	4	3.43	379.71064	0.06	0.03	0.06	0.05	0.13	-0.22	0.16	0.15	0.10	0.00	0.17	0.001	0.16	0.13	0.04	0.18	-0.02	0.24	0.269
17121	Gomphidic acid	2	0.90	395.038046	1.60	0.15	2.04	0.14	0.01	0.93	0.73	3.89	0.68	4.38	0.48	0.000	1.08	0.51	1.59	0.43	3.17	0.75	0.045
17420	2-Fluoro-5-nitrobenzaldehyde	2	0.99	402.05038	0.95	0.07	1.01	0.06	0.17	0.16	0.19	1.73	0.28	1.21	0.20	0.001	0.01	0.29	0.07	0.20	0.96	0.17	0.124
17735	3-O-Methylniveusin A	2	4.19	409.186197	0.98	0.08	1.17	0.07	0.03	0.04	0.35	1.39	0.39	1.11	0.26	0.001	0.20	0.33	0.67	0.24	1.03	0.36	0.119
17975	N-Docosahexaenoyl GABA	2	11.0	414.299272	0.22	0.04	0.19	0.04	0.21	2.30	0.68	3.75	0.91	4.77	1.08	0.000	1.20	0.46	2.99	0.40	3.67	0.73	0.000
17976	No identification	4	9.38	414.299307	0.39	0.04	0.32	0.10	0.41	1.32	0.35	2.05	0.75	2.28	0.76	0.000	1.36	0.84	2.06	1.61	2.74	2.63	0.034
17978	No identification	4	10.7	414.299357	0.18	0.03	0.20	0.06	0.56	3.27	0.42	4.01	0.64	4.80	0.60	0.000	2.06	0.45	3.83	0.76	5.28	1.18	0.000
18079	3b-Hydroxy-5-cholenoic acid	2	10.8	416.31502	0.53	0.05	0.39	0.08	0.38	2.20	0.85	2.41	0.53	3.03	0.70	0.004	2.00	0.39	2.75	0.39	2.02	0.44	0.170
18080	No identification	4	10.1	416.315105	0.45	0.05	0.51	0.12	0.61	0.80	0.54	1.74	0.40	1.82	0.55	0.004	1.40	0.16	1.87	0.38	1.53	0.46	0.345
18432	No identification	4	0.97	424.870921	1.74	0.11	1.81	0.11	0.03	-1.42	0.46	0.71	0.47	1.32	0.36	0.002	0.42	0.47	-0.87	0.43	-0.62	0.52	0.070
18524	6-(2-Carboxyethyl)-7-hydroxy-2,2-dimethyl-4-chromanone glucoside	2	9.38	427.158905	0.87	0.08	0.72	0.06	0.41	-0.08	0.12	-0.07	0.16	-0.46	0.14	0.001	-0.19	0.09	-0.23	0.17	-0.15	0.09	0.498
18731	No identification	4	1.26	431.223846	0.45	0.05	0.55	0.07	0.86	1.41	0.28	2.21	0.28	2.95	0.45	0.000	1.03	0.21	2.04	0.34	3.03	0.29	0.000
18771	Chenodeoxycholic acid glycine conjugate	2	9.93	432.309526	0.44	0.05	0.32	0.07	0.33	2.00	0.49	2.42	0.59	2.76	0.69	0.000	1.94	0.43	2.34	0.32	2.58	0.56	0.053
18773	No identification	4	9.38	432.309833	0.41	0.05	0.32	0.09	0.21	1.27	0.34	1.97	0.72	2.37	0.73	0.001	1.06	0.88	1.89	1.66	2.75	2.72	0.005

No	Identification	LI	Rt	Mass m/z	Median t0					Median iAUC NW							Median iAUC OB						
			min	Da	NW	sem	OB	sem	p	A	sem	B	sem	C	sem	FDR	A	sem	B	sem	C	sem	FDR
18863	Lithocholic acid glycine conjugate	2	12.7	434.32534	0.25	0.03	0.26	0.04	0.90	2.47	0.70	3.52	0.82	4.46	1.09	0.000	1.96	0.33	3.18	0.75	3.92	0.71	0.000
19200	Vismodegib	2	1.00	442.99655	0.42	0.07	0.60	0.13	0.46	0.94	0.47	1.73	0.54	2.28	0.37	0.003	-0.05	0.40	2.27	0.76	1.38	0.42	0.006
19403	No identification	4	10.1	448.304735	0.19	0.05	0.19	0.05	0.92	1.67	0.77	3.04	0.95	3.44	0.99	0.000	1.59	0.38	2.85	0.34	3.18	0.63	0.040
19448	No identification	4	1.04	450.037679	0.55	0.08	0.88	0.13	0.01	1.41	0.37	2.68	0.49	4.62	0.41	0.000	1.13	0.42	3.25	0.35	4.69	0.46	0.000
19482	Glycodeoxycholic acid	2	11.0	450.320662	0.19	0.04	0.17	0.04	0.32	2.04	0.72	3.61	0.99	4.93	1.17	0.000	1.12	0.46	2.97	0.41	3.70	0.77	0.000
19483	No identification	4	10.7	450.32078	0.17	0.03	0.21	0.05	0.45	3.07	0.41	3.97	0.66	4.58	0.63	0.000	2.02	0.45	3.66	0.80	5.15	1.24	0.000
19802	3,5-Dinitrosalicylate	2	0.88	457.008284	0.85	0.08	1.13	0.06	0.01	0.95	0.38	2.50	0.31	2.39	0.37	0.000	0.77	0.34	1.58	0.27	2.26	0.33	0.065
20003	Shoyuflavone C	2	0.90	463.025225	1.81	0.16	2.29	0.13	0.00	0.74	0.93	4.45	0.75	4.02	0.62	0.000	1.86	0.51	1.12	0.41	3.83	1.00	0.075
20006	3-Hydroxy-3-carboxymethyl-adipic acid	2	4.18	463.104548	1.24	0.11	1.35	0.13	0.08	-0.90	0.43	1.12	0.55	2.38	0.41	0.000	0.50	0.65	1.38	0.41	1.48	0.75	0.422
20194	Glycocholic acid	2	9.31	466.315452	0.22	0.04	0.23	0.07	0.83	2.18	0.41	3.18	0.58	4.09	0.74	0.000	1.78	0.47	2.64	0.84	2.93	1.09	0.001
21404	CD 1790 (HMDB0060809)	2	7.85	496.206725	0.52	0.05	0.41	0.07	0.14	-0.14	0.15	-0.81	0.14	-0.82	0.15	0.000	-0.43	0.18	0.00	0.14	-0.38	0.17	0.388
22247	Adenylylselenate	2	0.90	516.996921	1.88	0.23	2.09	0.15	0.07	1.52	1.11	4.68	1.03	6.28	0.94	0.002	2.62	0.95	1.96	0.57	4.04	1.00	0.314
22520	Azido-2',3'-dideoxyuridine	2	4.42	524.197507	1.06	0.17	1.78	0.33	0.00	-0.13	0.78	1.68	0.62	1.86	0.69	0.001	1.19	0.62	1.43	1.29	2.84	1.08	0.208
22560	No identification	4	0.88	524.99548	0.89	0.08	1.24	0.08	0.03	0.84	0.30	2.24	0.41	2.85	0.37	0.000	0.95	0.25	1.99	0.30	2.03	0.45	0.109
24023	No identification	4	0.88	570.98833	0.92	0.12	1.22	0.12	0.01	0.87	0.57	2.81	0.52	3.57	0.59	0.001	1.27	0.58	1.51	0.61	2.50	0.56	0.752
24419	No identification	4	0.88	586.96598	0.86	0.07	0.98	0.07	0.24	0.74	0.30	1.25	0.35	2.36	0.28	0.004	1.06	0.35	1.53	0.35	1.29	0.38	0.678
24731	No identification	4	10.7	599.248149	0.58	0.16	0.56	0.08	0.35	0.02	0.64	-0.80	0.52	-0.72	0.62	0.005	-1.10	0.24	-0.58	0.39	-0.69	0.35	0.592
24735	Lansimide 2	2	11.5	599.248824	0.84	0.18	0.92	0.13	0.47	-0.64	0.53	-1.89	0.63	-1.71	0.66	0.003	-1.21	0.29	-0.69	0.51	-0.63	0.39	0.559
24806	APC (HMDB0060661)	2	9.97	601.264041	1.18	0.31	0.89	0.14	0.12	-0.58	0.95	-1.77	1.23	-1.43	1.10	0.004	-1.39	0.38	-1.25	0.54	-1.85	0.65	0.731
25051	Debromoaplysiatoxin	2	10.2	610.357201	0.17	0.06	0.26	0.16	0.93	1.24	0.74	2.09	0.57	1.93	0.76	0.000	2.02	0.24	2.44	0.55	2.38	0.68	0.084
25193	No identification	4	10.4	616.255245	0.68	0.12	0.70	0.10	0.39	-0.22	0.44	-0.65	0.63	-1.01	0.42	0.002	-0.53	0.25	-0.69	0.28	-1.03	0.26	0.767
25265	Teraspiridole B_130089	2	8.23	619.274746	0.51	0.23	0.44	0.09	0.26	-0.04	1.13	-0.49	0.44	-1.05	0.57	0.003	-1.03	0.34	-0.49	0.29	-0.44	0.32	0.528
25422	Glycochenodeoxycholic acid 3-glucuronide	2	9.24	626.3521	0.32	0.08	0.34	0.21	0.78	1.03	0.27	1.40	0.33	1.27	0.43	0.000	0.63	0.21	1.37	0.21	1.65	0.28	0.000
25900	Norcisapride	2	1.25	649.230986	0.64	0.07	0.97	0.20	0.33	1.06	0.21	1.51	0.36	2.45	0.36	0.001	0.51	0.39	1.15	0.66	1.40	0.66	0.083
28733	Tribenuron-methyl	2	1.26	854.193255	1.77	0.14	3.02	0.23	0.00	-0.78	0.36	1.19	0.36	0.45	0.36	0.005	-0.37	0.43	-0.49	0.54	0.53	0.50	0.017

[1] Median values of the fasting and postprandial features. HF meals: A = 500, B = 1000, C = 1500 kcal; sem, standard error of mean; *p*, *p*-value of Kruskal–Wallis rank sum test without adjustment for multiple testing comparing the fasting values of the features in the NW and OB groups. Features with a significant fasting differences between the NW and OB group are indicated in yellow; *FDR*, FDR corrected *p*-value of Kruskal–Wallis rank sum test comparing the postprandial iAUC of the NW and OB group after the ingestion of each dose of the HF meal (Benjamini and Hochberg 1995). Abbreviations: iAUC: incremental area under the curve; LI: level of identification; NW: normal weight; OB: obese; Rt: retention time.

SUPPLEMENTAL TABLE S2 Correlation of 52 LC-MS features at fasting (t0) with iAUC results for HF meals A, B, and C for NW and OB groups and for HF meal A for the NW group only[1]

No	Feature with level 1 or 3 identification	Meal A NW+OB		Meal B NW+OB		Meal C NW+OB		Meal A NW only	
		r	*FDR*	*r*	*FDR*	*r*	*FDR*	*r*	*FDR*
1006		-0.30	0.12	-0.57	0.00	-0.30	0.17	-0.43	0.21
10249		-0.21	0.28	-0.28	0.12	0.18	0.41	-0.49	0.16
10564		-0.76	0.00	-0.77	0.00	-0.76	0.00	-0.76	0.01
12456		-0.46	0.02	-0.68	0.00	-0.71	0.00	-0.64	0.05
12793		-0.45	0.02	-0.38	0.04	-0.26	0.24	0.19	0.58
12804		-0.54	0.00	-0.35	0.05	-0.12	0.59	-0.60	0.08
12916		-0.22	0.25	-0.31	0.09	-0.10	0.64	-0.16	0.65
12953		-0.45	0.02	-0.31	0.10	-0.05	0.80	-0.66	0.05
13313		-0.70	0.00	-0.71	0.00	-0.60	0.00	-0.76	0.01
13414		-0.62	0.00	-0.42	0.02	-0.45	0.03	-0.60	0.08
13672		-0.86	0.00	-0.88	0.00	-0.94	0.00	-0.87	0.00
13726		-0.36	0.06	-0.60	0.00	-0.29	0.19	-0.36	0.30
14126	Allantoin	-0.15	0.42	-0.30	0.11	0.03	0.86	-0.11	0.72
1419		-0.27	0.16	-0.29	0.11	-0.10	0.64	-0.23	0.49
14622		-0.79	0.00	-0.87	0.00	-0.93	0.00	-0.71	0.03
14692		-0.36	0.06	-0.44	0.02	-0.41	0.05	-0.14	0.68
1544	Creatine	0.17	0.39	0.43	0.02	0.58	0.00	0.34	0.31
1659	Alanine	-0.48	0.01	-0.39	0.03	-0.53	0.01	-0.48	0.18
17121		-0.32	0.09	-0.45	0.02	-0.23	0.27	-0.36	0.30
17735		-0.39	0.04	-0.68	0.00	-0.51	0.01	-0.43	0.21
1797		-0.51	0.01	-0.53	0.00	-0.37	0.08	-0.46	0.19
18432		-0.57	0.00	-0.62	0.00	-0.67	0.00	-0.51	0.15
19448		-0.20	0.30	-0.04	0.84	0.11	0.62	-0.01	0.98
19802		-0.43	0.02	-0.38	0.04	-0.43	0.04	-0.43	0.21
20003		-0.34	0.08	-0.60	0.00	-0.29	0.19	-0.29	0.40
22520		-0.23	0.25	-0.25	0.17	0.06	0.76	-0.13	0.68
22560		-0.39	0.04	-0.37	0.05	-0.54	0.01	-0.24	0.48
24023		-0.70	0.00	-0.73	0.00	-0.50	0.01	-0.63	0.05
2518		-0.45	0.02	-0.56	0.00	-0.30	0.17	-0.42	0.22
2548	Lysine	-0.33	0.09	-0.30	0.10	-0.07	0.75	-0.36	0.30
28733		-0.02	0.93	-0.55	0.00	0.15	0.51	-0.30	0.38
2960		-0.70	0.00	-0.70	0.00	-0.54	0.01	-0.74	0.02
3632		-0.36	0.06	-0.55	0.00	-0.23	0.27	-0.66	0.05
3681		-0.49	0.01	-0.34	0.06	-0.23	0.27	-0.24	0.48
3852		-0.64	0.00	-0.47	0.01	-0.31	0.16	-0.55	0.12
3902		-0.40	0.04	-0.44	0.02	-0.25	0.24	-0.65	0.05
4315	Arginine	-0.26	0.18	-0.40	0.03	-0.25	0.25	-0.35	0.31

No	Feature with level 1 or 3 identification	Meal A NW+OB		Meal B NW+OB		Meal C NW+OB		Meal A NW only	
4740		-0.36	0.06	-0.51	0.01	-0.18	0.41	-0.45	0.21
4896		-0.71	0.00	-0.52	0.00	-0.40	0.06	-0.85	0.00
5270	Homoarginine	-0.27	0.17	-0.01	0.95	-0.32	0.15	-0.09	0.76
6369		-0.59	0.00	-0.59	0.00	-0.71	0.00	-0.58	0.09
66		-0.61	0.00	-0.43	0.02	-0.28	0.20	-0.52	0.14
6672		-0.12	0.51	0.14	0.45	0.59	0.00	0.51	0.14
7137		-0.14	0.45	-0.54	0.00	-0.37	0.08	-0.25	0.48
781	Valine	-0.49	0.01	-0.55	0.00	-0.26	0.24	-0.22	0.51
7876		-0.39	0.04	-0.37	0.04	-0.30	0.18	-0.51	0.14
858	Homoserine or threonine	-0.56	0.00	-0.49	0.01	-0.52	0.01	-0.26	0.46
867		-0.55	0.00	-0.37	0.05	-0.23	0.27	-0.31	0.38
8826		-0.52	0.01	-0.49	0.01	-0.13	0.57	-0.42	0.22
9144		0.00	0.99	-0.40	0.03	-0.25	0.24	-0.43	0.21
9539		-0.42	0.03	-0.63	0.00	-0.44	0.03	-0.39	0.26
9703		-0.29	0.14	-0.43	0.02	-0.04	0.83	-0.14	0.68

[1]HF meals: A = 500, B = 1000, C = 1500 kcal; NW, normal weight group; OB, obese group; *r*, Spearman correlation coefficient; Features with a significant correlations are indicated in yellow; *FDR*, FDR corrected *p*-value of Kruskal–Wallis rank sum test (Benjamini and Hochberg 1995).

SUPPLEMENTAL TABLE S3 Median results of ten amino acids quantified with GC-MS at fasting (t0) and iAUC for NW and OB subjects for meal A, B, and C and FDR corrected *p*-values[1]

Identification	Rt	Quantifier	Qualifier	Median fasting data (t0)					Median iAUC NW							Median iAUC OB						
	min	m/z	m/z	NW	sem	OB	sem	*p*	A	sem	B	sem	C	sem	*FDR*	A	sem	B	sem	C	sem	*FDR*
Beta-alanine	23.40	174	248	1.28E+05	1.52E+04	1.31E+05	2.17E+04	0.857	8.28E+05	1.30E+05	1.46E+06	2.15E+05	2.32E+06	1.30E+05	0.005	4.82E+05	6.15E+04	1.03E+06	1.37E+05	1.38E+06	2.32E+05	0.001
Cystine	34.05	218	146	4.93E+05	7.34E+04	5.12E+05	3.81E+04	0.563	2.38E+04	2.48E+05	2.22E+05	1.03E+05	4.23E+05	3.11E+05	0.613	3.20E+05	2.83E+05	-2.96E+04	1.87E+05	1.28E+05	1.89E+05	0.805
Isoleucine	20.19	158	218	3.63E+06	3.03E+05	5.00E+06	4.13E+05	0.001	5.52E+06	1.62E+06	1.27E+07	1.99E+06	2.31E+07	3.66E+06	0.006	3.54E+06	7.77E+05	1.42E+07	2.82E+06	1.54E+07	1.97E+06	0.018
Proline	20.40	142	216	8.37E+06	1.37E+06	1.04E+07	1.35E+06	0.065	2.36E+06	4.50E+06	8.05E+06	3.97E+06	3.00E+07	1.05E+07	0.160	-8.49E+05	3.53E+06	1.14E+07	6.95E+06	1.03E+07	5.32E+06	0.312
Threonine	22.4	218	291	3.24E+06	3.31E+05	2.92E+06	1.57E+05	0.539	1.86E+06	9.39E+05	4.03E+06	6.85E+05	9.08E+06	1.60E+06	0.014	6.38E+05	3.49E+05	2.41E+06	8.00E+05	3.01E+06	6.39E+05	0.097
Phenylalanine	26.92	218	192	3.67E+06	2.43E+05	3.82E+06	1.18E+05	0.294	1.12E+06	9.99E+05	2.68E+06	6.53E+05	7.92E+06	1.15E+06	0.027	-2.78E+05	6.59E+05	1.81E+06	4.18E+05	3.47E+06	5.02E+05	0.015
Ornithine	29.32	142	420	3.25E+06	1.58E+05	3.57E+06	1.81E+05	0.060	3.53E+06	6.96E+05	7.73E+06	3.32E+05	1.05E+07	2.85E+05	0.002	3.72E+06	5.79E+05	5.44E+06	6.13E+05	5.65E+06	9.04E+05	0.018
Histidine	30.53	154	254	3.46E+06	2.55E+05	3.68E+06	2.82E+05	0.329	3.49E+06	1.01E+06	5.37E+06	1.00E+06	9.86E+06	7.91E+05	0.016	1.10E+06	7.99E+05	3.05E+06	8.72E+05	4.24E+06	7.03E+05	0.058
Tyrosine	30.71	280	100	1.12E+06	4.64E+04	1.35E+06	6.08E+04	<0.001	2.26E+05	1.51E+05	1.55E+06	1.45E+05	2.28E+06	1.67E+05	0.002	-1.55E+05	3.20E+05	7.55E+05	1.80E+05	1.19E+06	1.83E+05	0.075
Glutamic acid	26.68	246	128	3.52E+05	4.03E+04	5.73E+05	7.06E+04	<0.001	-1.36E+05	1.91E+05	1.99E+05	2.17E+05	8.80E+05	1.49E+05	0.022	-2.05E+05	1.52E+05	1.25E+05	2.50E+05	2.07E+05	2.47E+05	0.519

[1] Median values: HF meals: A = 500, B = 1000, C = 1500 kcal; Rt, retention time; Quantifier, mass detector quantifier ion; Qualifier, mass detector qualifier ion m/z mass signal; NW, normal weight; OB, obese; sem, standard error of mean; *p*, *p*-value of Kruskal–Wallis rank sum test without adjustment for multiple testing; Features with a significant fasting differences between the NW and OB group are indicated in yellow; Features with a significant fasting differences between the NW and OB group are indicated in yellow; *FDR*, FDR corrected *p*-value of Kruskal–Wallis rank sum test (Benjamini and Hochberg 1995).

SUPPLEMENTAL TABLE S4 Characterization of the postprandial response (iAUC 0-6 h) of metabolic, hormonal, and inflammatory variables in NW (n = 15) and OB (n = 17) men having consumed 500, 1000, and 1500 kcal of the HF meal

Parameter	Mean iAUC NW			Mean iAUC OB		
	A	B	C	A	B	C
Glucose, mmol*h/L	-2.23 ± 0.42	-1.93 ± 0.72	-0.90 ± 0.43	-1.19 ± 0.53	-0.71 ± 0.64	1.75 ± 0.97*
Insulin mU*h/L	15.24 ± 3.83[c]	40.40 ± 6.49[b]	64.46 ± 10.49[a]	68.29 ± 11.14*[c]	180.06 ± 23.79*[b]	321.16 ± 45.03*[a]
Triglyceride mmol*h/L	2.11 ± 0.36[b]	4.65 ± 0.54[a]	5.26 ± 0.55[a]	2.44 ± 0.36[c]	4.87 ± 0.50[b]	5.98 ± 0.46[a]
Cholesterol, mmol*h/L	-1.05 ± 0.41	-1.25 ± 0.28	-1.28 ± 0.26	-0.43 ± 0.22	-0.14 ± 0.21*	-0.78 ± 0.20
HDL cholesterol, mmol*h/L	-0.325 ± 0.092[a]	-0.776 ± 0.084[b]	-0.698 ± 0.091[b]	-0.243 ± 0.089[a]	-0.615 ± 0.147[b]	-0.548 ± 0.084[b]
Total:HDL cholesterol h	0.380 ± 0.208[b]	1.383 ± 0.406[a]	1.200 ± 0.374[a]	0.371 ± 0.299[b]	2.277 ± 0.579[a]	1.773 ± 0.329[a]
CRP, mg*h/L	0.081 ± 0.236	-0.266 ± 0.122	-0.436 ± 0.084	-0.309 ± 0.162	-0.338 ± 0.246	-0.713 ± 0.517
IL-6, pg*h/mL	-11.99 ± 5.56[b]	9.81 ± 7.91[a]	9.47 ± 6.43[a]	-2.14 ± 5.53	9.90 ± 10.51	22.88 ± 6.76
Endotoxin, EU*h/mL	0.64 ± 0.45[a]	2.23 ± 0.62[b]	2.90 ± 0.64[b]	0.35 ± 0.78	1.15 ± 0.96	2.71 ± 0.44
GLP-1, pmol*h/L	45.29 ± 14.40	54.59 ± 18.87	75.13 ± 30.63	46.12 ± 7.98	68.53 ± 8.85	76.13 ± 12.85

Values are means ± SEMs. HF meals: A = 500, B = 1000, C = 1500 kcal; Means in a row without a common letter differ within a group (Kruskal Wallis rank-sum-test $p < 0.05$ and pairwise comparison Conover-Iman test $p < 0.05$). *Indicates a different mean from the corresponding normal-weight group (Wilcoxon Rank Sum Test, $p < 0.05$). Glucose, CRP, TGs, cholesterol, and HDL cholesterol were analyzed in plasma. Insulin, IL-6, endotoxin, and GLP-1 were analyzed in serum. CRP, C-reactive protein; EU, endotoxin units; GLP-1, glucagon-like peptide-1; HF, high-fat; iAUC, incremental AUC (Adapted from Schwander et al. 2014).

SUPPLEMENTAL TABLE S5 Correlation coefficients and FDR values of postprandial iAUC clinical data with 52 iAUC LC-MS results for NW subjects[1]

No	Feature	Total/HDL cholesterol		HDL cholesterol		Total cholesterol		Endotoxin		GLP-1		Glucose		CRP		IL-6		Insulin		TNF-alpha		Triglyceride	
		r	*FDR*	*r*	*FDR*	*r*	*FDR*	*r*	*FDR*	*r*	*FDR*	*r*	*FDR*	*r*	*FDR*	*r*	*FDR*	*r*	*FDR*	*r*	*FDR*	*r*	*FDR*
1006		0.18	0.31	-0.33	0.04	-0.31	0.34	0.13	0.44	0.21	0.67	0.05	0.97	-0.17	0.78	0.04	0.88	0.52	0.00	0.04	0.90	0.38	0.02
10249		0.25	0.19	-0.37	0.03	-0.25	0.34	0.44	0.04	0.01	0.98	0.29	0.41	-0.43	0.16	0.25	0.68	0.57	0.00	0.21	0.84	0.50	0.00
10564		0.02	0.91	0.18	0.27	0.17	0.43	-0.29	0.14	0.00	0.99	-0.24	0.77	0.48	0.11	-0.05	0.88	-0.02	0.90	-0.07	0.84	-0.08	0.61
12456		0.18	0.30	-0.10	0.54	-0.06	0.76	0.11	0.51	-0.08	0.84	-0.08	0.97	0.17	0.78	-0.15	0.88	0.10	0.53	-0.18	0.84	0.25	0.11
12793		0.27	0.18	-0.38	0.03	-0.08	0.64	0.26	0.18	0.17	0.67	0.06	0.97	-0.01	1.00	0.24	0.68	0.48	0.00	0.24	0.84	0.47	0.00
12804		0.23	0.21	-0.35	0.04	-0.24	0.34	0.16	0.35	0.18	0.67	0.03	0.97	-0.03	1.00	0.05	0.88	0.24	0.13	0.04	0.90	0.39	0.01
12916		0.21	0.26	-0.39	0.02	-0.28	0.34	0.17	0.32	0.10	0.84	0.11	0.97	-0.15	0.86	-0.04	0.88	0.31	0.05	0.01	0.95	0.32	0.04
12953		0.25	0.19	-0.38	0.03	-0.22	0.36	0.40	0.09	0.03	0.89	0.13	0.97	-0.21	0.78	0.08	0.88	0.43	0.01	0.12	0.84	0.44	0.01
13313		0.13	0.43	-0.19	0.25	0.02	0.92	0.17	0.32	0.27	0.67	0.22	0.85	0.11	0.96	0.18	0.86	0.31	0.05	0.14	0.84	0.28	0.07
13414		0.13	0.44	-0.36	0.03	-0.18	0.42	0.34	0.13	0.10	0.84	-0.17	0.97	0.01	1.00	0.03	0.89	0.37	0.02	0.09	0.84	0.34	0.03
13672		-0.17	0.34	0.40	0.02	0.09	0.63	-0.28	0.15	-0.19	0.67	-0.49	0.03	0.27	0.56	-0.24	0.68	-0.26	0.09	-0.26	0.84	-0.24	0.11
13726		0.21	0.26	-0.38	0.03	-0.23	0.34	0.22	0.23	0.20	0.67	0.03	0.97	-0.11	0.96	0.09	0.88	0.46	0.00	0.08	0.84	0.49	0.00
14126	Allantoin	0.31	0.14	-0.45	0.01	-0.33	0.34	0.55	0.00	-0.06	0.85	0.39	0.10	-0.39	0.16	0.30	0.68	0.48	0.00	0.29	0.84	0.46	0.00
1419		0.29	0.15	-0.43	0.02	-0.23	0.34	0.28	0.14	0.18	0.67	-0.07	0.97	-0.09	0.96	0.09	0.88	0.43	0.01	0.13	0.84	0.50	0.00
14622		0.31	0.14	0.07	0.65	0.16	0.43	-0.04	0.78	-0.08	0.84	-0.38	0.10	0.40	0.16	0.05	0.88	-0.02	0.91	0.04	0.90	0.24	0.11
14692		0.19	0.28	-0.37	0.03	-0.23	0.34	0.24	0.20	0.07	0.85	0.11	0.97	-0.10	0.96	0.21	0.68	0.43	0.01	0.21	0.84	0.39	0.01
1544	Creatine	0.42	0.10	-0.56	0.00	-0.31	0.34	0.47	0.03	0.03	0.89	0.38	0.10	-0.34	0.24	0.29	0.68	0.63	0.00	0.27	0.84	0.63	0.00
1659	Alanine	-0.09	0.57	-0.16	0.32	-0.09	0.64	0.09	0.56	0.26	0.67	0.01	0.98	-0.07	1.00	0.13	0.88	0.46	0.00	0.07	0.84	0.20	0.19
17121		0.32	0.14	-0.41	0.02	-0.09	0.64	0.13	0.42	0.18	0.67	0.07	0.97	0.00	1.00	0.23	0.68	0.52	0.00	0.18	0.84	0.48	0.00
17735		0.26	0.19	-0.28	0.08	-0.01	0.98	0.15	0.36	0.06	0.85	-0.07	0.97	0.06	1.00	0.00	0.98	0.42	0.01	-0.01	0.95	0.34	0.03
1797		0.16	0.35	-0.33	0.05	-0.26	0.34	0.22	0.23	0.28	0.67	0.04	0.97	-0.18	0.78	0.04	0.88	0.56	0.00	0.05	0.90	0.42	0.01
18432		0.16	0.35	-0.17	0.29	-0.17	0.43	0.25	0.19	-0.05	0.86	0.11	0.97	-0.26	0.56	0.17	0.87	0.25	0.11	0.14	0.84	0.27	0.08
19448		0.25	0.19	-0.32	0.05	-0.16	0.43	0.33	0.13	0.24	0.67	0.39	0.10	-0.30	0.41	0.29	0.68	0.50	0.00	0.28	0.84	0.54	0.00
19802		0.19	0.28	-0.30	0.06	-0.10	0.62	0.30	0.14	0.06	0.85	-0.05	0.97	0.04	1.00	0.15	0.88	0.44	0.01	0.14	0.84	0.41	0.01
20003		0.20	0.28	-0.41	0.02	-0.29	0.34	0.18	0.32	0.21	0.67	0.14	0.97	-0.24	0.63	0.04	0.88	0.46	0.00	0.05	0.90	0.44	0.01
22520		0.31	0.14	-0.24	0.14	-0.08	0.64	0.15	0.36	0.03	0.89	-0.10	0.97	-0.05	1.00	-0.24	0.68	0.34	0.03	-0.24	0.84	0.32	0.04
22560		0.23	0.21	-0.42	0.02	-0.18	0.43	0.29	0.14	0.17	0.67	-0.04	0.97	-0.08	1.00	0.04	0.88	0.50	0.00	0.07	0.84	0.43	0.01
24023		-0.01	0.96	-0.10	0.53	-0.15	0.44	0.26	0.18	0.03	0.89	0.14	0.97	-0.38	0.16	0.23	0.68	0.40	0.01	0.15	0.84	0.33	0.03
2518		0.19	0.29	-0.31	0.05	-0.26	0.34	0.28	0.14	0.24	0.67	0.04	0.97	-0.15	0.86	0.09	0.88	0.59	0.00	0.07	0.84	0.45	0.00
2548	Lysine	0.31	0.14	-0.45	0.01	-0.21	0.37	0.29	0.14	0.18	0.67	-0.05	0.97	-0.05	1.00	0.10	0.88	0.45	0.00	0.15	0.84	0.51	0.00
28733		0.31	0.14	-0.40	0.02	-0.30	0.34	0.17	0.32	0.09	0.84	0.10	0.97	-0.01	1.00	0.15	0.88	0.34	0.03	0.13	0.84	0.39	0.01
2960		0.37	0.10	-0.49	0.01	-0.28	0.34	0.35	0.13	0.15	0.73	-0.07	0.97	-0.19	0.78	0.19	0.84	0.51	0.00	0.21	0.84	0.57	0.00
3632		0.24	0.20	-0.33	0.04	-0.18	0.43	0.18	0.32	0.20	0.67	0.03	0.97	-0.12	0.96	0.06	0.88	0.53	0.00	0.04	0.90	0.45	0.00
3681		0.28	0.17	-0.32	0.05	-0.12	0.54	0.21	0.23	0.13	0.81	0.01	0.98	-0.03	1.00	0.09	0.88	0.29	0.06	0.08	0.84	0.38	0.02
3852		0.29	0.15	-0.35	0.04	-0.16	0.43	0.17	0.33	0.10	0.84	-0.05	0.97	0.10	0.96	0.11	0.88	0.30	0.05	0.10	0.84	0.36	0.02

No	Feature	Total/HDL cholesterol		HDL cholesterol		Total cholesterol		Endotoxin		GLP-1		Glucose		CRP		IL-6		Insulin		TNF-alpha		Triglyceride	
		r	*FDR*	*r*	*FDR*	*r*	*FDR*	*r*	*FDR*	*r*	*FDR*	*r*	*FDR*	*r*	*FDR*	*r*	*FDR*	*r*	*FDR*	*r*	*FDR*	*r*	*FDR*
3902		0.30	0.14	-0.46	0.01	-0.24	0.34	0.31	0.13	0.08	0.84	-0.11	0.97	-0.06	1.00	0.07	0.88	0.43	0.01	0.09	0.84	0.51	0.00
4315	Arginine	0.37	0.10	-0.46	0.01	-0.23	0.35	0.32	0.13	0.13	0.81	-0.12	0.97	-0.09	0.96	0.11	0.88	0.43	0.01	0.13	0.84	0.56	0.00
4740		0.14	0.42	-0.31	0.05	-0.20	0.37	0.22	0.23	0.28	0.67	0.06	0.97	-0.17	0.78	0.11	0.88	0.55	0.00	0.10	0.84	0.39	0.01
4896		0.25	0.19	-0.37	0.03	-0.28	0.34	0.24	0.20	0.24	0.67	0.00	0.98	-0.18	0.78	-0.01	0.98	0.38	0.01	-0.02	0.95	0.50	0.00
5270	Homo-arginine	0.25	0.19	-0.41	0.02	-0.22	0.36	0.24	0.19	0.11	0.84	-0.18	0.97	-0.09	0.96	0.02	0.94	0.47	0.00	0.03	0.94	0.42	0.01
6369		0.23	0.21	-0.32	0.05	-0.21	0.36	0.18	0.32	-0.05	0.86	-0.13	0.97	-0.04	1.00	0.01	0.97	0.24	0.13	-0.02	0.94	0.38	0.02
66		0.20	0.28	-0.29	0.06	-0.12	0.54	0.29	0.14	0.21	0.67	-0.05	0.97	-0.12	0.96	0.08	0.88	0.23	0.14	0.09	0.84	0.30	0.05
6672		0.35	0.12	-0.37	0.03	-0.13	0.54	0.36	0.13	0.10	0.84	0.29	0.41	-0.38	0.16	0.24	0.68	0.56	0.00	0.19	0.84	0.52	0.00
7137		0.31	0.14	-0.39	0.02	-0.24	0.34	0.36	0.13	0.11	0.84	-0.07	0.97	-0.17	0.78	0.14	0.88	0.43	0.01	0.14	0.84	0.56	0.00
781	Valine	0.35	0.12	-0.38	0.03	-0.12	0.54	0.33	0.13	-0.09	0.84	-0.02	0.97	-0.01	1.00	0.13	0.88	0.43	0.01	0.10	0.84	0.50	0.00
7876		0.10	0.52	-0.32	0.05	-0.25	0.34	0.25	0.18	0.17	0.67	-0.05	0.97	-0.05	1.00	0.12	0.88	0.33	0.03	0.17	0.84	0.35	0.03
858	Homoserine or Threonine	0.25	0.19	-0.43	0.02	-0.31	0.34	0.30	0.14	0.08	0.84	0.08	0.97	-0.21	0.78	0.22	0.68	0.45	0.00	0.20	0.84	0.51	0.00
867		0.29	0.15	-0.32	0.05	-0.13	0.52	0.21	0.23	0.16	0.73	0.02	0.97	0.00	1.00	0.11	0.88	0.29	0.06	0.10	0.84	0.41	0.01
8826		0.45	0.10	-0.54	0.00	-0.17	0.43	0.32	0.13	0.15	0.73	0.00	0.98	0.01	1.00	0.17	0.87	0.33	0.04	0.19	0.84	0.53	0.00
9144		0.38	0.10	-0.31	0.05	0.00	0.99	0.04	0.78	0.06	0.85	-0.14	0.97	-0.06	1.00	-0.06	0.88	0.18	0.25	-0.04	0.90	0.34	0.03
9539		0.12	0.47	-0.25	0.11	-0.19	0.42	0.25	0.18	0.18	0.67	0.05	0.97	-0.19	0.78	0.11	0.88	0.46	0.00	0.09	0.84	0.39	0.01
9703		0.40	0.10	-0.46	0.01	-0.19	0.42	0.32	0.13	0.08	0.84	0.05	0.97	0.00	1.00	0.08	0.88	0.38	0.01	0.10	0.84	0.51	0.00

[1]Feature, feature with level 1 and 3 identification; *r*, Spearman correlation coefficient; Features with a significant correlations are indicated in yellow*; FDR*, FDR corrected *p*-value (Benjamini and Hochberg 1995); GLP-1, glucagon-like peptide-1; CRP, C-reactive protein; IL-6, interleukin-6.
